# Alterations in peripheral blood NK cell subsets and function in patients with HBeAg-positive chronic hepatitis B during pregnancy

**DOI:** 10.3389/fcimb.2025.1657367

**Published:** 2025-10-06

**Authors:** Xiaokun Shen, Xiaosong Zhang, Yanqiu Cao, Li Zhang, Hongxia Yuan, Haixin Wang, Yarui Zhou, Shuo Diao, Xingshu Qi, Fujie Li, Qingjie Fan, Shinan Li

**Affiliations:** ^1^ Department of Immunology, School of Basic Medical Science, Jinzhou Medical University, Jinzhou, China; ^2^ Collaborative Innovation Center for Age-related Disease, Jinzhou Medical University, Jinzhou, China; ^3^ Department of Neonatology, Maternity and Children’s Hospital, Jinzhou, China; ^4^ Department of Clinical Laboratory, The First Affiliated Hospital of Jinzhou Medical University, Jinzhou, China; ^5^ Department of Infectious Diseases, The First Affiliated Hospital of Jinzhou Medical University, Jinzhou, China

**Keywords:** pregnancy, chronic hepatitis B, NK, HBeAg, IFN-γ, Th17

## Abstract

**Background:**

The majority of patients with chronic hepatitis B (CHB) are in the immune-tolerant phase during pregnancy, exhibiting relatively stable liver disease. However, some hepatitis B e antigen (HBeAg)-positive pregnant women may develop liver dysfunction, a condition with an unclear pathogenesis.

**Methods:**

In this study, we analyzed the phenotype and function of natural killer (NK) cell subsets using flow cytometry and enzyme-linked immunosorbent assay in HBeAg-positive pregnant women, HBeAg-negative pregnant women, and healthy pregnant controls.

**Results:**

We found that HBeAg-positive pregnant women exhibited a decreased proportion of peripheral blood CD56bright NK cells, which correlated negatively with HBV DNA loads and alanine transaminase (ALT) levels, whereas an increased proportion of CD56^dim^ NK cells correlated positively with HBV DNA loads and ALT levels. CD56^dim^ NK cells in HBeAg-positive women displayed a highly activated phenotype characterized by elevated expression of activating receptors (NKG2D and CD226) and reduced expression of inhibitory receptors (NKG2A and CD158b). Consistent with this phenotype, their CD56^dim^ NK cells demonstrated enhanced cytotoxic capacity by diminished interferon-γ production and enhanced CD107a and granzyme-B production. Furthermore, NK cells from HBeAg-positive pregnant women failed to suppress Th17 cell polarization. This study elucidates alterations in peripheral blood NK cell subsets, phenotypes, and functions in pregnant women with CHB. Collectively, these results indicate that peripheral NK cells in HBeAg-positive pregnant women exhibit a unique profile of activation coexisting with functional impairment.

## 1 Introduction

Hepatitis B virus (HBV) infection remains a major global public health problem and a leading cause of chronic liver disease, cirrhosis, and hepatocellular carcinoma (Easterbrook et al., 2024). In pregnant women, HBV infection carries profound implications for both maternal and child health. For the mother, it is associated with an increased risk of adverse outcomes, including gestational diabetes, intrahepatic cholestasis of pregnancy, and preterm labor (Pawlowska, 2024). For the child, the paramount concern is mother-to-child transmission (MTCT), which represents the primary route of HBV transmission globally and is a major driver of chronic infection (Joshi and Coffin, 2020). In HBV infection without preventive interventions, the risk of MTCT is particularly high, reaching 70% to 90% among mothers who are positive for both hepatitis B surface antigen (HBsAg) and hepatitis B e antigen (HBeAg). Even HBeAg-negative (HBeAg^-^) mothers carry a significant, albeit lower (10% to 40%), transmission risk (Tran, 2016). Although most pregnant women with chronic hepatitis B (CHB) are in the immune-tolerant (IT) phase and experience a stable disease with minimal progression or hepatic failure during pregnancy, the dynamic immunologic shifts of pregnancy and postpartum can trigger disease activity. Notably, some women experience hepatitis flare-ups, particularly within 12 weeks postpartum (Zhang et al., 2022a). Mothers positive for both hepatitis B surface antigen (HBsAg) and HBeAg face the highest risk. Despite this risk, the mechanisms underlying HBV disease activity during pregnancy remain complex and incompletely understood.

Natural killer (NK) cells are critical innate immune effectors with a complex, dual role during pregnancy, needing to defend against pathogens while maintaining tolerance to the semi-allogeneic fetus. Human NK cells are broadly categorized into two subsets based on CD16 (FcγRIIIα) and CD56 (NCAM1) expression: CD56^bright^CD16^-^ NK cells, which are primarily responsible for cytokine secretion and enhance broader immune responses (macrophage activation and antigen presentation), and CD56^dim^CD16^+^ NK cells, which mediate cytotoxicity via perforin/granzyme release and antibody-dependent cellular cytotoxicity to destroy infected cells opsonized by antiviral IgG antibodies (Cooper et al., 2001; Michel et al., 2016). Activated NK cells, especially CD56^bright^CD16^-^, can secrete potent antiviral cytokines such as IFN-γ and TNF-α, which directly limit viral replication (Moretta et al., 2002), and CD56^dim^CD16^+^ NK cells exert direct cytotoxicity via receptors such as TRAIL, NKG2D, and Fas-L or through cytolytic granule release (Glassner et al., 2012). Tissue-specific NK cells can play a critical role in maintaining tissue homeostasis. Decidual CD56^bright^ NK (dNK) cells, which largely express CD49a or CD27, accumulate in the maternal decidua during pregnancy (Hanna et al., 2006). A previous study revealed that local dNK cells are impaired and fail to inhibit Th17 cell expansion in patients who experience recurrent spontaneous abortions (RSAs) (Fu et al., 2013). TGF‐β is released by CD56^bright^CD25^+^ dNK cells and is essential for immune tolerance during early human pregnancy (Tao et al., 2015). However, the immunomodulatory functions of NK cell subsets are poorly characterized in HBV-infected pregnant women.

Th17 cells exert strong proinflammatory effects through cytokines such as IL-17A, IL-17F, IL-21, and IL-22, and proinflammatory Th17 cells play a negative role in pregnancy (Lentz et al., 2022). Elevation of Th17 levels in human peripheral blood and the decidua has been detected in patients with RSA (Fu et al., 2013). Their frequencies, along with those of effector molecules (e.g., IL-17A and IL-22), increase with the severity of chronic liver diseases (Zhang et al., 2020) and contribute to CHB progression. Notably, the frequency of IFN-γ^+^IL-17^+^ Th17 cells correlates positively with HBV DNA load, alanine transaminase (ALT) levels, HBeAg/HBsAg titers, and cytokine (IL-6 and IL-17) levels (Xie et al., 2022). IL-17 levels also appear to increase in pregnant women compared with non-pregnant women (Plug et al., 2025). A well-controlled level of IL-17 appears to be required for a successful pregnancy. Although Th17 cells participate in the immune response to HBV, the specific mechanisms regulating their number and function, especially in the context of HBV during pregnancy, remain unclear.

In this study, we aimed to understand the alterations in the distribution, phenotype, and function of NK cell subsets in HBeAg-positive (HBeAg^+^) pregnant women. Focusing on the comparison of changes in NK cell subsets and differences in functional Th17 cells among HBeAg^+^ pregnant women, HBeAg^-^ pregnant women, and healthy pregnant women, we characterized how NK cell subsets participate in the regulation of Th17 functions.

## 2 Materials and methods

### 2.1 Ethics statement

Our study was conducted in accordance with the guidelines of the Declaration of Helsinki and the principles of good clinical practice. All patients and healthy controls who participated in this study provided written informed consent, and our research was approved by the ethics committee of the First Affiliated Hospital of Jinzhou Medical University, China.

### 2.2 Study subjects

According to the diagnostic criteria of chronic hepatitis B and asymptomatic HBV carrier formulated by the Ministry of Health of China, pregnant women with hepatitis B, pregnant women with HBV carrier stage, and healthy pregnant women who underwent physical examination from the laboratory and infectious disease departments of the hospital were selected (all signed an informed consent form). The subjects were tested for 10 immune tests, liver function, and HBV-DNA before the experiment. Superinfection of viral hepatitis A, C, D, and E, fatty liver disease, alcoholic liver disease, autoimmune liver disease, and drug-induced hepatitis were excluded. Peripheral blood samples were collected from 30 pregnant women with hepatitis B (HBsAg^+^, HBeAg^+^), 28 pregnant women with HBV carrier period (HBsAg^+^, HBeAg^-^), and 14 healthy pregnant women for subsequent cell phenotype, function, and *in vitro* culture experiments. The clinical characteristics are summarized in **Table 1**, and detailed clinical information is shown in **Supplementary Table S1**.

**Table 1:** Clinical characteristics of the study population.

Variable	HC PW (*N*=14)	HBeAg^-^ PW (*N*=28)	HBeAg^+^ PW (*N*=30)	*P*-value (HC vs. HBeAg^-^)	*P*-value (HC vs. HBeAg^+^)	*P*-value (HBeAg^-^ vs. HBeAg^+^)
Age (years)	25 (21–31)	24.5 (19–34)	25.4 (20–37)	0.4862	0.2811	0.7779
Gestational age (weeks)	25 (10–32)	24 (13–35)	26 (11–31)	0.5390	0.9950	0.1984
ALT (U/L)	24 (19–42)	29.61 (18–39)	81.53 (45–134)	0.0169*	<0.0001****	<0.0001****
HBV-DNA (IU/mL)	N.D.	1.41 × 10^4^ (<1,000–6.97 × 10^4^)	1.28 × 10^8^ (5.5 × 10^6^–6.18 × 10^8^)	**-**	**-**	<0.0001****
HBsAg (IU/mL)	N.D.	5,123.84 (161.01–18,934.65)	41,786 (632.6–102,141.68)	–	–	<0.0001****
HBsAb (IU/L)	>10	<10	<10	–	–	–
HBeAg (IU/mL)	N.D.	0.037 (0.001–0.153)	427.9 (28.14–1,439.67)	–	–	<0.0001****
HBeAb (COI)	N.D.	0.04 (0.002–0.916)	5.3 (0.4–7.89)	–	–	<0.0001****
HBcAb (COI)	N.D.	<1	<1	–	–	–

All of the data in the table are described by “mean value”, and each value range is marked. *P*<0.05 was considered statistically significant.

N.D., not detected. *P<0.05; ****P<0.0001.

### 2.3 Isolation of human peripheral blood mononuclear cells

The collected venous blood was centrifuged, and the upper plasma was separated and frozen in the refrigerator at -80°C. The lower blood cells were diluted with 1× PBS, and the fresh peripheral blood mononuclear cells (PBMCs) were separated by density gradient centrifugation after Ficoll lymphocyte isolation solution was added. The isolated PBMCs were washed twice with 1× PBS, the PBMCs were suspended with RPMI-1640 complete medium supplemented with 10% FBS and 1% penicillin–streptomycin solution (PS), and cell counts were performed.

### 2.4 Flow cytometric analysis

The PBMCs (1 × 10^6^ cells) of pregnant women in different groups were stained using two antibody panels:— panel A: FITC anti-human CD16 (BD Pharmingen, clone B73.1), PE anti-human CD158b (BD Pharmingen, clone CH-L), Alexa Fluor^®^ 647 anti-human CD56 (BD Pharmingen, clone B159), and APC-Cy7 anti-human CD3 (BD Pharmingen, clone SK7) and panel B: FITC anti-human CD226 (BD Pharmingen, clone DX11), PE anti-human CD159a (NKG2A) (Biolegend, clone S19004C), Alexa Fluor^®^ 647 anti-human CD56 (BD Pharmingen, clone B159), PE/Cy7 anti-human CD314 (NKG2D) (BD Pharmingen, clone 1D11), and APC-Cy7 anti-human CD3 (BD Pharmingen, clone SK7). These were incubated at 4°C for 30 min in the dark. Cells were harvested after centrifugation and washed twice with 1× PBS. Finally, 200 μL 1×PBS cells were added, and the expression of NK cell surface receptors were detected by flow cytometry.

### 2.5 Detection of the effector function of NK cells

PBMCs (1 ×10^6^ cells/mL) were added into 24-well culture plates for culture, and IL-2 (100 U/mL) was added to promote the normal growth of NK cells. The 24-well plates were placed in an incubator at 37°C and 5% CO_2_ for overnight culture. PMA (50 ng/mL), ionomycin (1 μg/mL), and anti-CD107a (BD Pharmingen, clone H4A3) were added to the culture for 3 h, and then moenomycin (1 μg/mL) was added to block for 1 h, and these were washed once with 1× PBS. After the resuspension of PBMCs with 100 uL of 1× PBS, human Fc receptor blocker was added and blocked for 30 min at room temperature. APC-Cy7 anti-human-CD3 (BD Pharmingen, clone SK7) and Alexa Fluor^®^ 647 anti-human-CD56 (NCAM1) (BD Pharmingen, clone B159) and corresponding isotype control antibodies were added to sample tubes and isotype tubes, respectively, and labeled at 4°C for 30m in in the dark. After cleaning with 1× PBS, 100 μL fixing solution was added to each tube and fixed at 4°C for 30 min. After fixation, 1× transmembrane liquid was added into each tube, mixed, and centrifuged at 6,000 rpm and 4°C for 2 min. After centrifugation, the supernatant was discarded, and 100 uL of 1× penetrating solution was added to resuspended PBMC. Anti-human-IFN-γ or anti-human-TNF-α and corresponding isotype control antibody were added to the sample tubes and isotype tubes, respectively, and labeled at 4°C for 60 min in the dark. The cells were washed twice with 500 uL transmembrane solution, respectively. Finally, the cells were suspended with 1× PBS and detected by flow cytometry.

### 2.6 NK cell sorting

PBMC suspensions from pregnant women with hepatitis B, pregnant women with HBV carrier, and healthy pregnant women were collected into sample tubes and stained with PerCP-Cy5.5-anti-Human-CD3 (BD Pharmingen, clone SK7) and Alexa Fluor^®^ 647 anti-human CD56 (NCAM1) (BD Pharmingen, clone B159), respectively. The fluorescently stained cells were separated by electrical deflection, and the sorted NK cells continued to be cultured.

### 2.7 NK cell culture

CD3^-^CD56^+^ NK cells sorted by flow cytometry as described above were seeded in 24-well plates with approximately 1 × 10^6^ cells/well, and 1 mL RPMI1640 medium containing 10% FBS and 1% PS was added to each well. IL-2 (100 U/mL), IL-12 (10 ng/mL), and IL-18 (10 ng/mL) were added into each sample well for stimulation culture and placed in an incubator at 37°C with 5% CO_2_. After 48 h of cytokine stimulation, the cells were collected to detect the expression of IFN-γ and other functional molecules by ELISA.

### 2.8 Cytokine ELISA

The plasma samples and NK cell culture supernatant were prepared, and the concentrations of IFN-γ and IL-17 in the samples were detected by using LEGEND MAX™ Human IFN-γ ELISA Kit and LEGEND MAX™ Human IL-17A ELISA Kit, respectively.

### 2.9 MACS purify CD4^+^ Th0 cells

After the total number of PBMCs was determined, they were washed once with 1× PBS, and 1 × 10^7^ cells were resuspended in 40 uL MACS buffer, followed by 10 μL antibody, and labeled at 4°C for 10 min in the dark. Next, 30 μL MACS buffer and 20 μL beads were added into the system, which was thoroughly mixed and labeled at 4°C for 15 min in the dark. In addition, 2 mL buffer was added and cleaned once. Before using the LD column, the separation column was rinsed with 500 μL buffer and placed on a 15-mL centrifuge tube. The cell precipitates were re-suspended with 500 μL buffer, and the cell suspensions were placed on the column. Finally, T cells were collected by washing three times with buffer, 500 uL each time, and after passing through the separation column. The T cells obtained from the abovementioned sorting were counted, and then the purity of T cells was determined by flow cytometry. If the purity was ≥95%, follow-up experiments were conducted.

### 2.10 Th17 cell polarization assay

PBMCs from pregnant women were isolated, and CD3^+^CD4^+^ T cells were purified by MACS. The concentration of isolated cells was adjusted to 1 × 10^6^/mL to prepare the plate. At 1 day in advance, 10 μg/mL human anti-CD3 was added to 96-well round-bottom sterile plates. These were placed at 4°C in a refrigerator, and the coated plates were incubated overnight. On the next day, the liquid was sucked out of the 96-well plate. The number of cells inoculated in each well was 1 × 10^5^ cells. The culture system was supplemented to 200 μL/well, and the polarizing cytokines IL-1β (10 ng/mL) and IL-23 (10 ng/mL) were added into the culture system. At the same time, 1 μg/mL anti-human CD3 and anti-human CD28 antibody were added, and the culture was polarized under cytokine stimulation. The experimental group was stimulated with IFN-γ, that is, the supernatant of CD3^-^CD56^+^ NK cells from pregnant women with hepatitis B and the concentration of cytokine IFN-γ (10 ng/mL) were added. The supernatant of CD3^-^CD56^+^ NK cells from healthy pregnant women and IFN-γ neutralizing antibody were added into the control group. After adding to the samples, the samples were placed in an incubator containing 5% CO_2_ at 37°C for 5 to 6 days.

### 2.11 Plasma ALT level detection assay

Following the instructions of the ALT test kit, the ALT test reagent was taken out and preheated at room temperature, and the standard reagent was dissolved; 10 μL plasma samples and standards were added to the microplate, respectively, and one well was reserved as blank control. ALT detection reagent at 200 μL was added into each well, and the OD value was determined with the wavelength of 340 nm by the enzyme-labeled instrument. The standard curve was drawn according to the concentration of the standard substance, and the plasma ALT levels of the samples to be measured were calculated respectively.

### 2.12 Detection of HBV-DNA load by quantitative PCR

According to the instructions of HBV-DNA real-time fluorescence quantitative PCR detection kit, HBV-DNA plasmid was used as the template to make the standard curve. Fluorescence groups were added to the PCR reaction system, and the whole PCR process was monitored in real time by the accumulation of fluorescence signals. Finally, the standard curve was used to quantitatively analyze the tested samples.

### 2.13 Statistical analysis

Statistical analysis of experimental data was performed using GraphPad Prism 9.0 software, and specific data was represented by “mean ± standard error”. The nonparametric Mann–Whitney *U*-test was used to evaluate the inter-group differences of HC-PW, HBeAg^+^-PW, and HBeAg^–^PW groups, and the intra-group differences were evaluated using paired *t*-test, and Spearman correlation was used for correlation analysis. *P*<0.05 was considered statistically significant (*P<0.05; **P<0.01; ***P<0.001; ns (not significant), *P* > 0.05).

## 3 Results

### 3.1 The distribution of NK cell subsets was altered in HBeAg^+^ pregnant women

**Figure 1:**
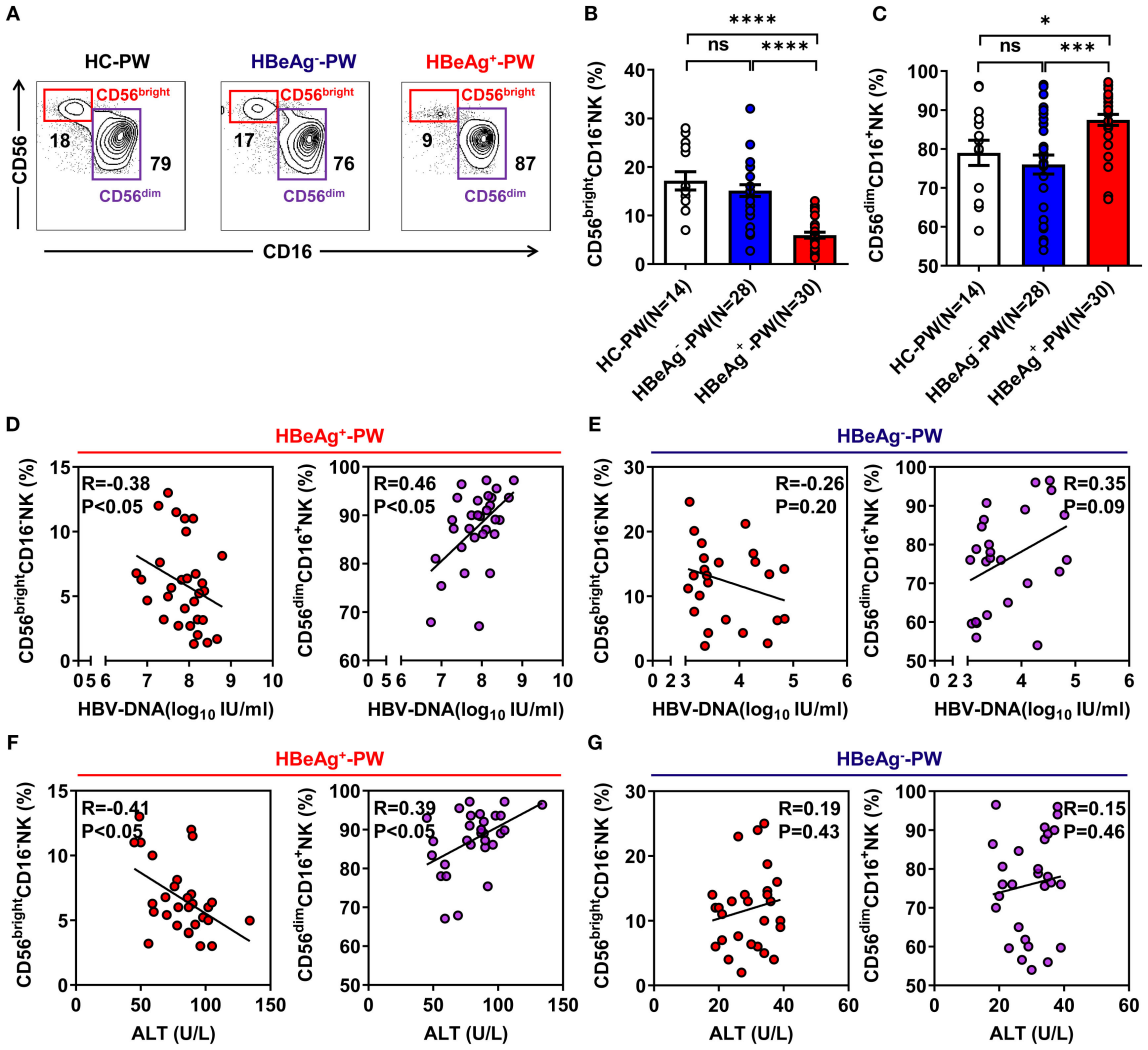
Subsets of NK cells were altered in the blood of HBeAg^+^ pregnant women. **(A)** Flow cytometric analysis of CD56 and CD16 expression by CD3^-^CD56^+^ NK cells after the application of a total lymphocyte gate. The values indicate the percentages of CD56^bright^CD16^-^ NK and CD56^dim^CD16^+^ NK cell subsets in the peripheral blood of healthy pregnant women, HBeAg^-^ pregnant women, and HBeAg^+^ pregnant women. **(B, C)** Pooled data showing the frequencies of CD56^bright^CD16^-^ NK and CD56^dim^CD16^+^ NK cells in healthy pregnant women (*N*=14), HBeAg^-^ pregnant women (*N*=28), and HBeAg^+^ pregnant women (*N*=30). **(D, E)** Correlation between CD56^bright^CD16^-^ NK subset or CD56^dim^CD16^+^ NK subset proportion and HBV-DNA load in HBeAg^+^ or HBeAg^-^ pregnant women. **(F, G)** Correlation between the proportion of CD56^bright^CD16^-^ NK subsets or CD56^dim^CD16^+^ NK subsets and ALT level in HBeAg^+^ or HBeAg^-^ pregnant women. Correlations between the two variables were evaluated using the Spearman rank correlation test. R, correlation coefficient; *P*-values are shown. Each dot represents one subject. HC-PW, healthy control pregnant women; HBeAg^–^PW, HBeAg^-^ pregnant women; HBeAg^+^-PW, HBeAg^+^ pregnant women. Unpaired *t*-test was used to compare two independent groups. *P<0.05; ***P<0.001; ****P<0.0001; n.s., not significant.

To determine whether the proportions of CD56^bright^ NK cells and CD56^dim^ NK cells were altered in HBeAg^+^ pregnant women, we isolated peripheral blood mononuclear cells (PBMCs) from HBeAg^+^, HBeAg^-^, and healthy pregnant women and analyzed the proportion of NK cells in each group by flow cytometry (the gating strategy is shown in **Supplementary Figures S1A, B**). We found that compared with those of healthy pregnant women, the changes in NK cell subsets in the peripheral blood of pregnant women with HBV mainly occurred in HBeAg^+^ pregnant women, which was primarily manifested as a decrease in the proportion of CD56^bright^ NK cells and an increase in the proportion of CD56^dim^ NK cells (**Figure 1A**; **Supplementary Figure S1C**). The statistical results showed that the percentage of CD56^bright^ NK cell subsets in HBeAg^+^ pregnant women was significantly lower than that in healthy pregnant women and HBeAg^-^ pregnant women (**Figure 1B**), whereas the proportion of CD56^dim^ NK cell subsets in HBeAg^+^ pregnant women was significantly higher (**Figure 1C**). The proportion of CD56^bright^ NK cells in HBeAg^+^ pregnant women was significantly negatively correlated with HBV viral loads (**Figure 1D**) and ALT levels (**Figure 1F**). In contrast, the proportion of CD56^bright^ NK cells of HBeAg^-^ pregnant women was not correlated with HBV viral loads (**Figure 1E**) or ALT levels (**Figure 1G**). The proportion of CD56^dim^ NK cells in HBeAg^+^ pregnant women was significantly positively correlated with HBV viral loads (**Figure 1D**) and ALT levels (**Figure 1F**). In contrast, the proportion of CD56^dim^ NK cells in HBeAg^-^ pregnant women was not correlated with HBV viral loads (**Figure 1E**) or ALT levels (**Figure 1G**). These findings indicate that NK subset alterations, particularly reduced CD56^bright^ subsets and increased CD56^dim^ subsets, are associated with hepatitis B severity during pregnancy.

### 3.2 The inhibitory receptors NKG2A and CD158b were downregulated on CD56^dim^ NK subsets but upregulated on CD56^bright^ NK cells in HBeAg^+^ pregnant women

**Figure 2:**
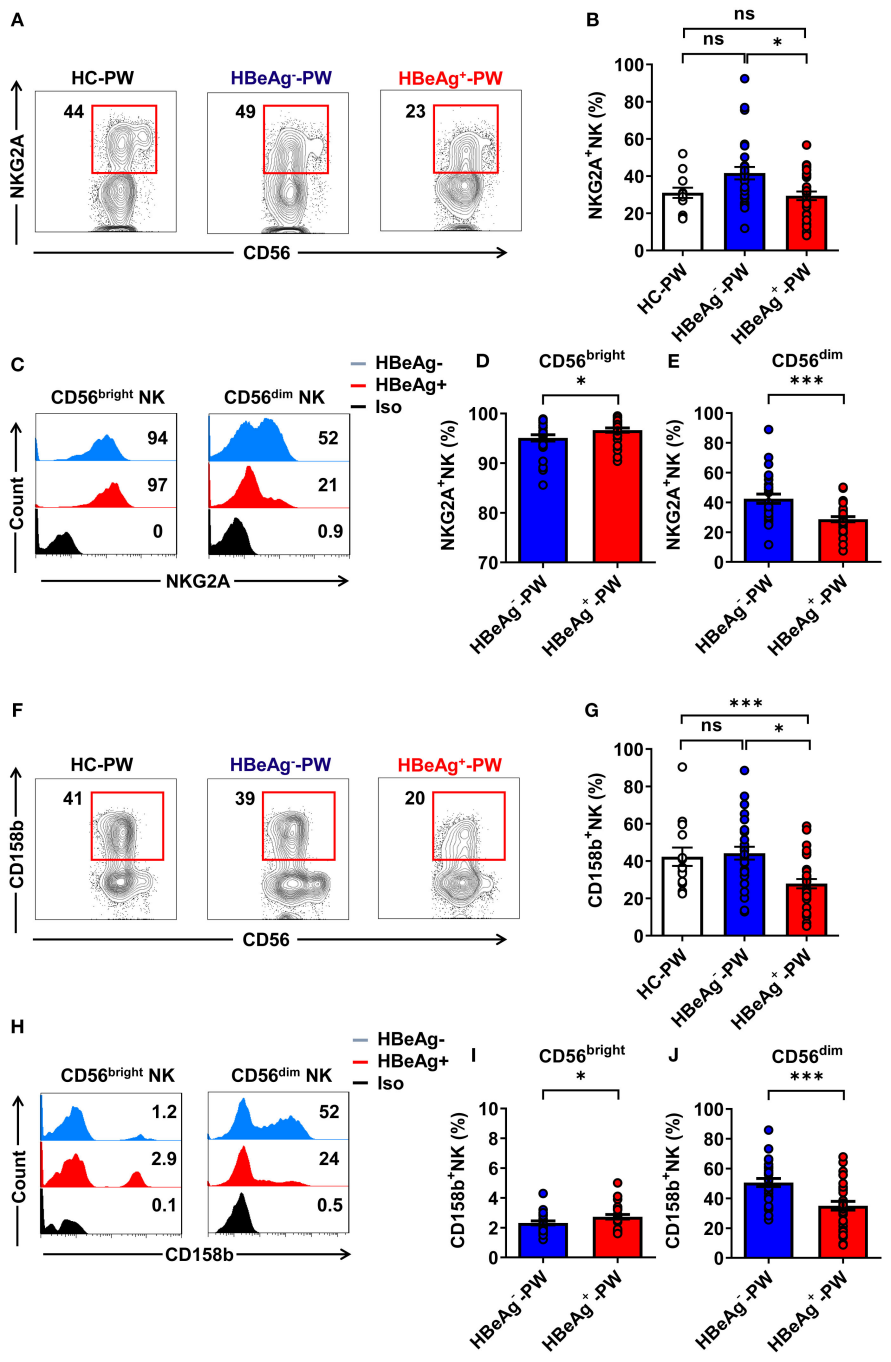
CD56^bright^ NK cells displayed a suppressive status while CD56^dim^ NK cells displayed a non-suppressive status in HBeAg^+^ pregnant women. **(A)** Flow cytometric analysis of NKG2A expression by all CD3^-^CD56^+^ NK cells in the peripheral blood of healthy pregnant women, HBeAg^-^ pregnant women, and HBeAg^+^ pregnant women. **(B)** Pooled data showing the frequencies of NKG2A^+^ NK cells in healthy pregnant women (*N*=14), HBeAg^-^ pregnant women (*N*=28), and HBeAg^+^ pregnant women (*N*=30). **(C)** Flow cytometric analysis of NKG2A expression by CD56^bright^ and CD56^dim^ NK cells in HBeAg^-^ and HBeAg^+^ pregnant women. **(D, E)** Pooled data showing the frequencies of NKG2A^+^CD56^bright^ NK and NKG2A^+^CD56^dim^ NK cells in HBeAg^-^ and HBeAg^+^ pregnant women. **(F)** Flow cytometric analysis of CD158b expression by all CD3^-^CD56^+^ NK cells in the peripheral blood of healthy pregnant women, HBeAg^-^ pregnant women, and HBeAg^+^ pregnant women. **(G)** Pooled data showing the frequencies of CD158b^+^ NK cells in healthy pregnant women (*n*=14), HBeAg^-^ pregnant women (*n*=28), and HBeAg^+^ pregnant women (*n*=30). **(H)** Flow cytometric analysis of CD158b expression by CD56^bright^ and CD56^dim^ NK cells of HBeAg^-^ and HBeAg^+^ pregnant women. **(I, J)** Pooled data showing the frequencies of CD158b^+^CD56^bright^ NK and CD158b^+^CD56^dim^ NK cells in HBeAg^-^ and HBeAg^+^ pregnant women. Unpaired *t*-test was used to compare two independent groups. *P<0.05; ***P<0.001; n.s., not significant.

When we further analyzed the phenotype of NK cells, we detected the inhibitory receptors (NKG2A and CD158b) on circulating NK cells from HBeAg^+^ pregnant women, HBeAg^-^ pregnant women, and healthy pregnant women (the gating strategy is shown in **Supplementary Figures S2A, B**). We found that the proportion of all NKG2A^+^ NK cells of HBeAg^+^ pregnant women was significantly lower than that of HBeAg^-^ pregnant women (**Figures 2A, B**), and there was a significant upregulation of inhibitory receptor NKG2A on CD56^bright^ NK subsets in HBeAg^+^ pregnant women compared with HBeAg^-^ pregnant women (**Figures 2C, D**). However, NKG2A on CD56^dim^ NK subsets was significantly downregulated in HBeAg^+^ pregnant women compared with HBeAg^-^ pregnant women (**Figures 2C, E**). Interestingly, we observed that the proportion of CD158b^+^ NK cells in HBeAg^+^ pregnant women was significantly reduced compared with that of healthy pregnant women and HBeAg^-^ pregnant women (**Figures 2F, G**). The CD158b expression levels were upregulated on CD56^bright^ NK subsets in HBeAg^+^ pregnant women compared with HBeAg^-^ pregnant women (**Figures 2H, I**), while CD158b expression was further downregulated on CD56^dim^ NK subsets in HBeAg^+^ pregnant women compared with HBeAg^-^ pregnant women (**Figures 2H, J**). These results suggest that in HBeAg^+^ pregnant women, CD56^dim^ NK cells display a non-suppressive status, whereas CD56^bright^ NK cells display a suppressive status.

### 3.3 The activated receptors CD226 and NKG2D were upregulated on CD56^dim^ NK cells in HBeAg^+^ pregnant women

**Figure 3:**
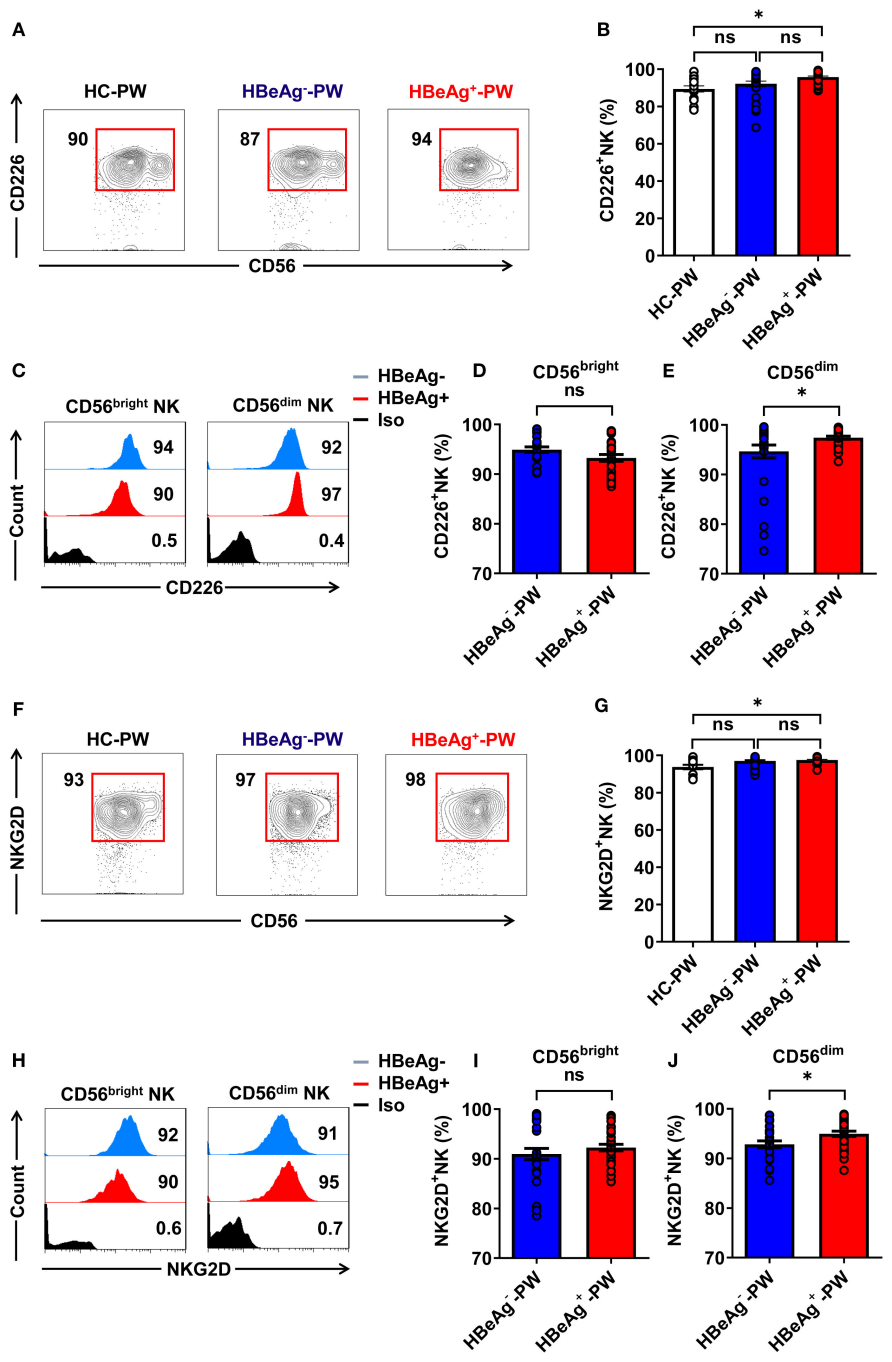
CD56^dim^ NK cells displayed an increased activation status in HBeAg^+^ pregnant women. **(A)** Flow cytometric analysis of CD226 expression by all CD3^-^CD56^+^ NK cells in the peripheral blood of healthy pregnant women, HBeAg^-^ pregnant women, and HBeAg^+^ pregnant women. **(B)** Pooled data showing the frequencies of CD226^+^ NK cells in healthy pregnant women (*N*=14), HBeAg^-^ pregnant women (*N*=28), and HBeAg^+^ pregnant women (*N*=30). **(C)** Flow cytometric analysis of CD226 expression by CD56^bright^ and CD56^dim^ NK cells of HBeAg^-^ and HBeAg^+^ pregnant women. **(D, E)** Pooled data showing the frequencies of CD226^+^CD56^bright^ NK and CD226^+^CD56^dim^ NK cells in HBeAg^-^ and HBeAg^+^ pregnant women. **(F)** Flow cytometric analysis of NKG2D expression by all CD3^-^CD56^+^ NK cells in the peripheral blood of healthy pregnant women, HBeAg^-^ pregnant women, and HBeAg^+^ pregnant women. **(G)** Pooled data showing the frequencies of NKG2D^+^ NK cells in healthy pregnant women (*N*=14), HBeAg^-^ pregnant women (*N*=28), and HBeAg^+^ pregnant women (*N*=30). **(H)** Flow cytometric analysis of NKG2D expression by CD56^bright^ and CD56^dim^ NK cells of HBeAg^-^ and HBeAg^+^ pregnant women. **(I, J)** Pooled data showing the frequencies of NKG2D ^+^CD56^bright^ NK and NKG2D ^+^CD56^dim^ NK cells in HBeAg^-^ and HBeAg^+^ pregnant women. Unpaired *t*-test was used to compare two independent groups. *P<0.05; n.s., not significant.

We detected the activated receptors (CD226 and NKG2D) on circulating NK cells (the gating strategy is shown in **Supplementary Figures 3A, B**), finding that the proportion of all CD226^+^ NK cells in HBeAg^+^ pregnant women was significantly higher than that in healthy pregnant women but not significantly different than that of HBeAg^-^ pregnant women (**Figures 3A, B**). However, further analysis revealed that CD226 on CD56^dim^ NK subsets was slightly increased in HBeAg^+^ pregnant women compared with HBeAg^-^ pregnant women (**Figures 3C, E**), while there was no difference in CD226 on CD56^bright^ NK subsets between HBeAg^+^ pregnant and HBeAg^-^ pregnant women (**Figures 3C, D**). Further analysis showed that the proportion of all NKG2D^+^ NK cells of HBeAg^+^ pregnant women was also significantly higher than that of healthy pregnant women (**Figures 3F, G**). Moreover, although NKG2D on CD56^dim^ NK subsets was increased in HBeAg^+^ pregnant women compared with HBeAg^-^ pregnant women (**Figures 3H, J**), there was no difference in NKG2D on CD56^bright^ NK subsets between HBeAg^+^ pregnant and HBeAg^-^ pregnant women (**Figures 3H, I**). Our findings showed that the activating receptors (CD226 and NKG2D) were upregulated while the inhibitory receptors (NKG2A and CD158b) were downregulated on CD6^dim^ NK subsets, suggesting the enhanced function of CD56^dim^ NK cells in HBeAg^+^ pregnant women.

### 3.4 IFN-γ secretion by all NK, CD56^bright^ NK, and CD56^dim^ NK cells was decreased in HBeAg^+^ pregnant women

**Figure 4:**
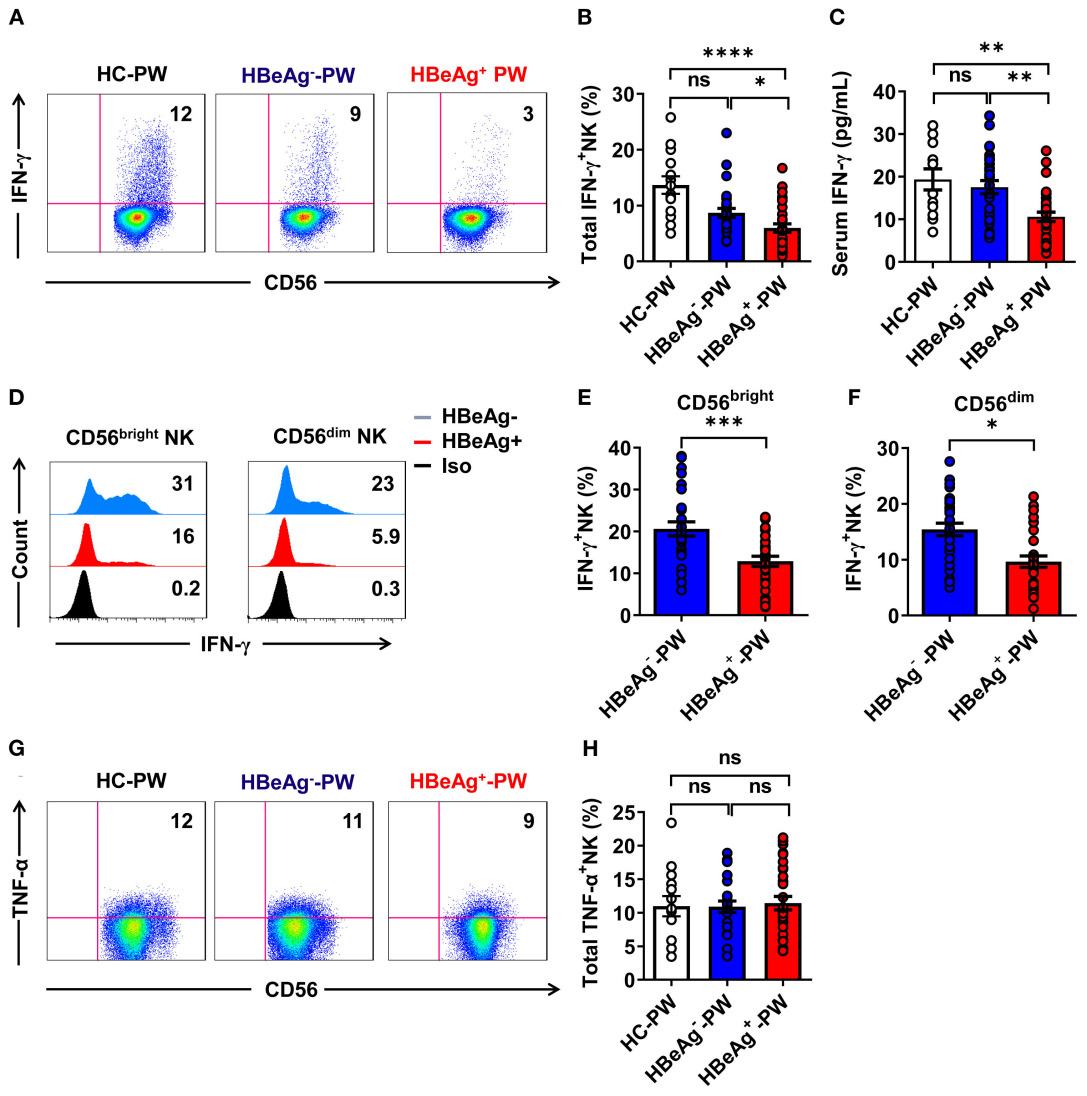
IFN-γ production by all NK cells was impaired in HBeAg^+^ pregnant women. **(A)** Representative dot plots represent IFN-γ expression by all NK cells from healthy pregnant women, HBeAg^-^ pregnant women, and HBeAg^+^ pregnant women. **(B)** Pooled data show the proportion of IFN-γ^+^ NK cells in healthy pregnant women, HBeAg^-^ pregnant women, and HBeAg^+^ pregnant women. **(C)** The plasma IFN-γ levels in the three groups are shown. **(D)** Flow cytometric analysis of IFN-γ expression by CD56^bright^ and CD56^dim^ NK cells of HBeAg^-^ and HBeAg^+^ pregnant women. **(E, F)** Pooled data showing the frequencies of IFN-γ^+^CD56^bright^ NK and IFN-γ^+^CD56^dim^ NK cells in HBeAg^-^ and HBeAg^+^ pregnant women. **(G)** Representative dot plots represent TNF-α expression by all NK cells from healthy pregnant women, HBeAg^-^ pregnant women, and HBeAg^+^ pregnant women. **(H)** Pooled data show the proportion of TNF-α^+^ NK cells in healthy pregnant women, HBeAg^-^ pregnant women, and HBeAg^+^ pregnant women. Unpaired *t*-test was used to compare two independent groups. *P<0.05; **P<0.01; ***P<0.001; ****P<0.0001; n.s., not significant.

To verify that the function of NK cells was enhanced, we detected IFN-γ secretion by NK cells in each group by flow cytometry and measured the plasma IFN-γ levels by enzyme-linked immunosorbent assay (ELISA). When we compared the differences in the production of IFN-γ by NK cells in different groups (the gating strategy is shown in **Supplementary Figure S4**), we found that the IFN-γ secreted by all NK cells in HBeAg^+^ pregnant women was significantly lower than that in HBeAg^-^ pregnant women and healthy pregnant women (**Figures 4A, B**). The plasma IFN-γ level of HBeAg^+^ pregnant women was significantly lower than that of HBeAg^-^ pregnant women and healthy pregnant women (**Figure 4C**). An analysis of the expression of IFN-γ on CD56^bright^ and CD56^dim^ NK cell subsets of HBeAg^+^ pregnant women and HBeAg^-^ pregnant women revealed that IFN-γ production by both CD56^bright^ and CD56^dim^ NK cell subsets was at low levels in HBeAg^-^ pregnant women (**Figures 4D–F**). However, we found no significant differences in TNF-α expression by all NK cells among the three groups (**Figures 4G, H**). These results demonstrate that alterations in NK cell subsets are also associated with IFN-γ functional impairment in HBeAg^+^ pregnant women.

### 3.5 NK cells from HBeAg^+^ pregnant women displayed increased cytolytic activity

**Figure 5:**
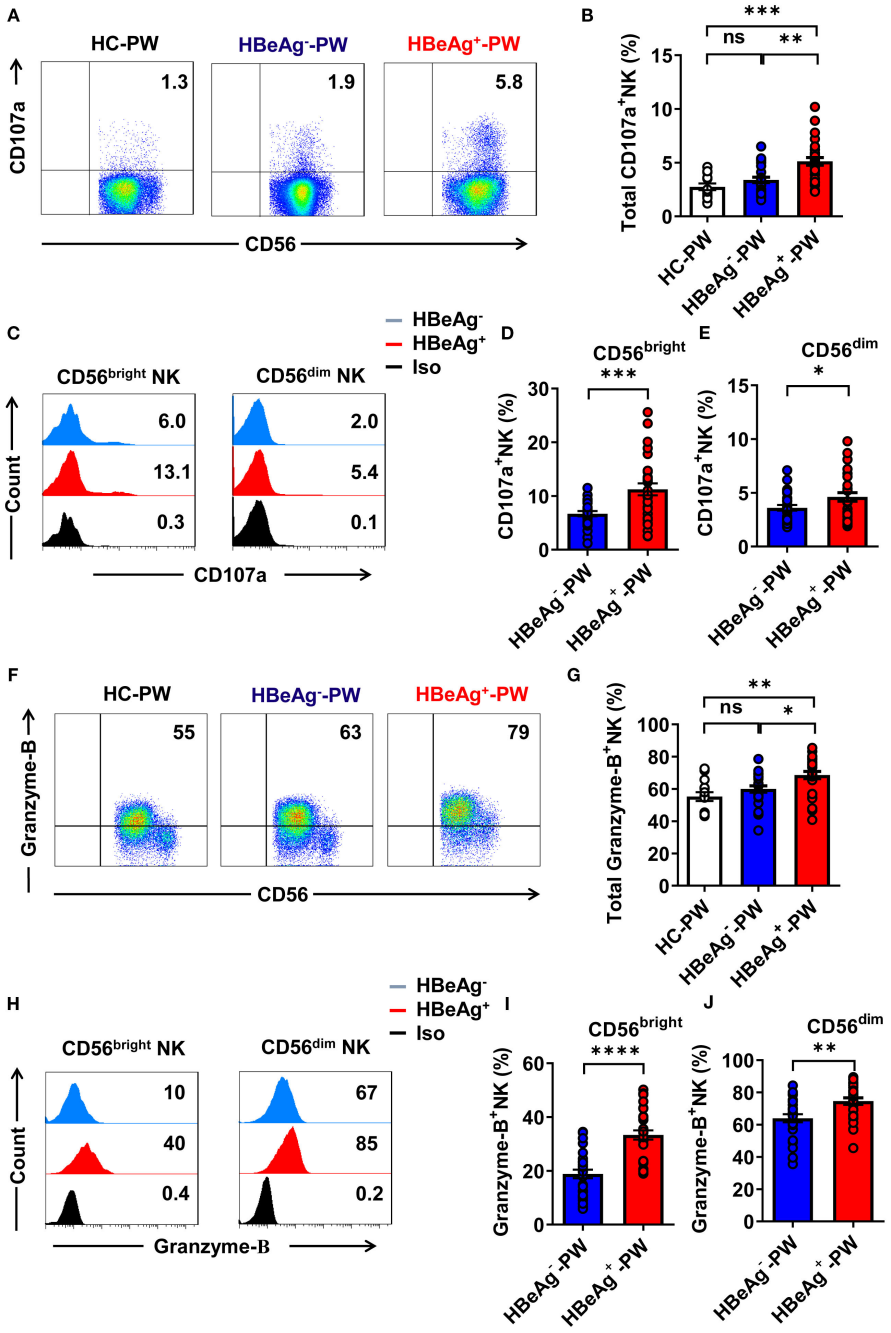
The cytotoxic activity of all NK cells was enhanced in HBeAg^+^ pregnant women. **(A)** Representative dot plots represent CD107a expression by all NK cells from healthy pregnant women, HBeAg^-^ pregnant women, and HBeAg^+^ pregnant women. **(B)** Pooled data show the proportion of CD107a^+^ NK cells in healthy pregnant women, HBeAg^-^ pregnant women, and HBeAg^+^ pregnant women. **(C)** Flow cytometric analysis of CD107a expression by CD56^bright^ and CD56^dim^ NK cells of HBeAg^-^ and HBeAg^+^ pregnant women. **(D, E)** Pooled data showing the frequencies of CD107a^+^CD56^bright^ NK and CD107a^+^CD56^dim^ NK cells in HBeAg^-^ and HBeAg^+^ pregnant women. **(F)** Representative dot plots represent granzyme-B expression by all NK cells from healthy pregnant women, HBeAg^-^ pregnant women, and HBeAg^+^ pregnant women. **(G)** Pooled data showing the proportion of granzyme-B^+^ NK cells in healthy pregnant women, HBeAg^-^ pregnant women, and HBeAg^+^ pregnant women. **(H)** Flow cytometric analysis of granzyme-B expression by CD56^bright^ and CD56^dim^ NK cells of HBeAg^-^ and HBeAg^+^ pregnant women. **(I, J)** Pooled data showing the frequencies of granzyme-B^+^CD56^bright^ NK and granzyme-B^+^CD56^dim^ NK cells in HBeAg^-^ and HBeAg^+^ pregnant women. Unpaired *t*-test was used to compare two independent groups. *P<0.05; **P<0.01; ***P<0.001; ****P<0.0001; n.s., not significant.

Detection of CD107a expression by all NK cells using flow cytometry showed that compared with healthy pregnant women and HBeAg^-^ pregnant women, the proportion of CD107a^+^ NK cells was significantly increased in HBeAg^+^ pregnant women (**Figures 5A, B**). An analysis of the expression of CD107a on CD56^bright^ and CD56^dim^ NK cell subsets in HBeAg^+^ pregnant women and HBeAg^-^ pregnant women revealed that CD107a expression on both CD56^bright^ and CD56^dim^ NK cell subsets was at higher levels in HBeAg^+^ pregnant women (**Figures 5C–E**). Detection of granzyme-B secretion by all NK cells in each group using flow cytometry showed (the gating strategy is shown in **Supplementary Figure S5**) that granzyme-B production in HBeAg^+^ pregnant women was significantly higher than that in HBeAg^-^ pregnant women and healthy pregnant women (**Figures 5F, G**). An analysis of the production of granzyme-B by CD56^bright^ and CD56^dim^ NK cell subsets revealed that granzyme-B production by both CD56^bright^ and CD56^dim^ NK cell subsets was at higher levels in HBeAg^+^ pregnant women than in HBeAg^-^ pregnant women and healthy pregnant women (**Figures 5H–J**). These results suggest that NK cells from HBeAg^+^ pregnant women display increased cytolytic activity.

### 3.6 The proportion of Th17 cells in the peripheral blood and the level of IL-17 in the plasma of HBeAg^+^ pregnant women significantly increased

**Figure 6:**
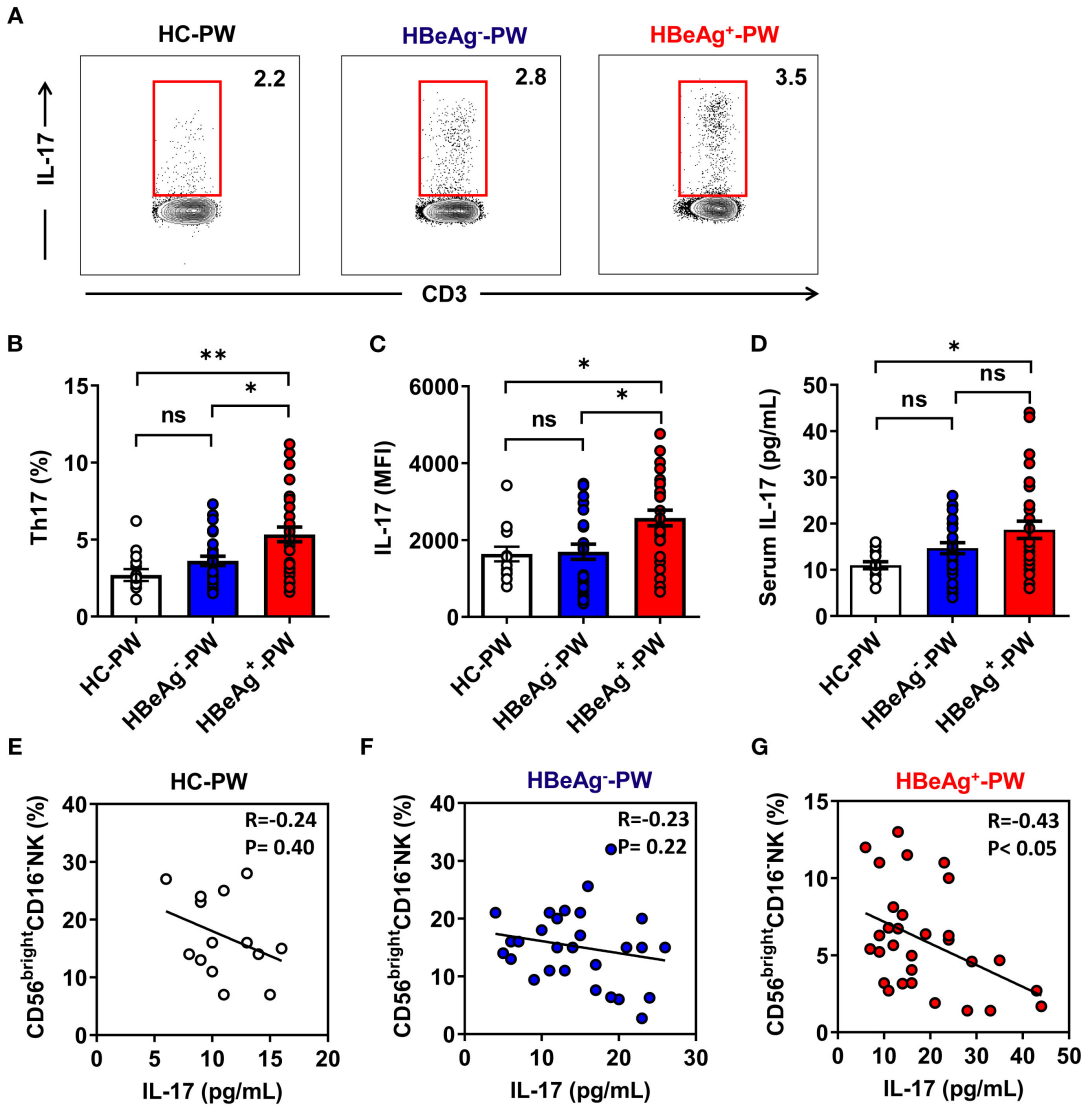
Th17-cell-derived IL-17 were enhanced in HBeAg^+^ pregnant women. **(A)** The proportion of Th17 cells in PBMCs of healthy pregnant women, HBeAg^-^ pregnant women, and HBeAg^+^ pregnant women was detected by flow cytometry. **(B)** Pooled data show the proportion of Th17 cells in healthy pregnant women, HBeAg^-^ pregnant women, and HBeAg^+^ pregnant women. **(C)** Pooled data showing the MFI of IL-17 on Th17 cells in healthy pregnant women, HBeAg^-^ pregnant women, and HBeAg^+^ pregnant women. **(D)** Plasma IL-17 levels in the three groups are shown. **(E–G)** Correlation between the proportion of CD56^bright^ CD16^-^ NK subsets and IL-17 level in healthy pregnant women, HBeAg^-^ pregnant women, and HBeAg^+^ pregnant women. Correlations between two variables were evaluated using the Spearman rank correlation test. R, correlation coefficient; *P*-values are shown. Each dot represents one subject. Unpaired *t*-test was used to compare two independent groups. *P<0.05; **P<0.01; n.s., not significant.

The results above indicate that the NK cell subsets of HBeAg^+^ pregnant women change at the same time the ability to secrete Th1 cytokines weakens, which mainly manifests as decreased expression of IFN-γ and level of serum IFN-γ. These findings suggest that the exacerbation of hepatitis in HBeAg^+^ pregnant women is accompanied by changes in NK cell subsets and function, which affect the function of other immune cells. To address whether these changes could affect the function of Th17 cells, we analyzed Th17 cells in HBeAg^+^ pregnant women by detecting the proportion of peripheral Th17 cells by flow cytometry (**Figure 6A**). Compared with that of healthy pregnant women, the proportion of Th17 cells in HBeAg^+^ pregnant women was significantly increased (**Figure 6B**), but the proportion did not significantly differ between HBeAg^-^ pregnant women and healthy pregnant women. The mean fluorescence intensity (MFI) of IL-17 in the Th17 cells of HBeAg^+^ pregnant women was significantly higher than that of healthy pregnant women and HBeAg^-^ pregnant women (**Figure 6C**). The plasma IL-17 level in HBeAg^+^ pregnant women was significantly higher than that in healthy pregnant women and HBeAg^-^ pregnant women (**Figure 6D**). An analysis of the correlation between the proportion of CD56^bright^CD16^-^ NK cells and plasma IL-17 levels showed that the proportion of CD56^bright^CD16^-^ NK cells in HBeAg^-^ pregnant women and healthy pregnant women was not correlated with plasma IL-17 levels (**Figures 6E, F**). In contrast, the proportion of CD56^bright^ CD16^-^ NK cells in HBeAg^+^ pregnant women was negatively correlated with plasma IL-17 levels (**Figure 6G**). These results further demonstrate a potential relationship between changes in NK cell subsets and Th17 cells in HBeAg^+^ pregnant women.

### 3.7 NK cells derived from HBeAg^+^ pregnant women could not inhibit Th17 cell polarization

**Figure 7:**
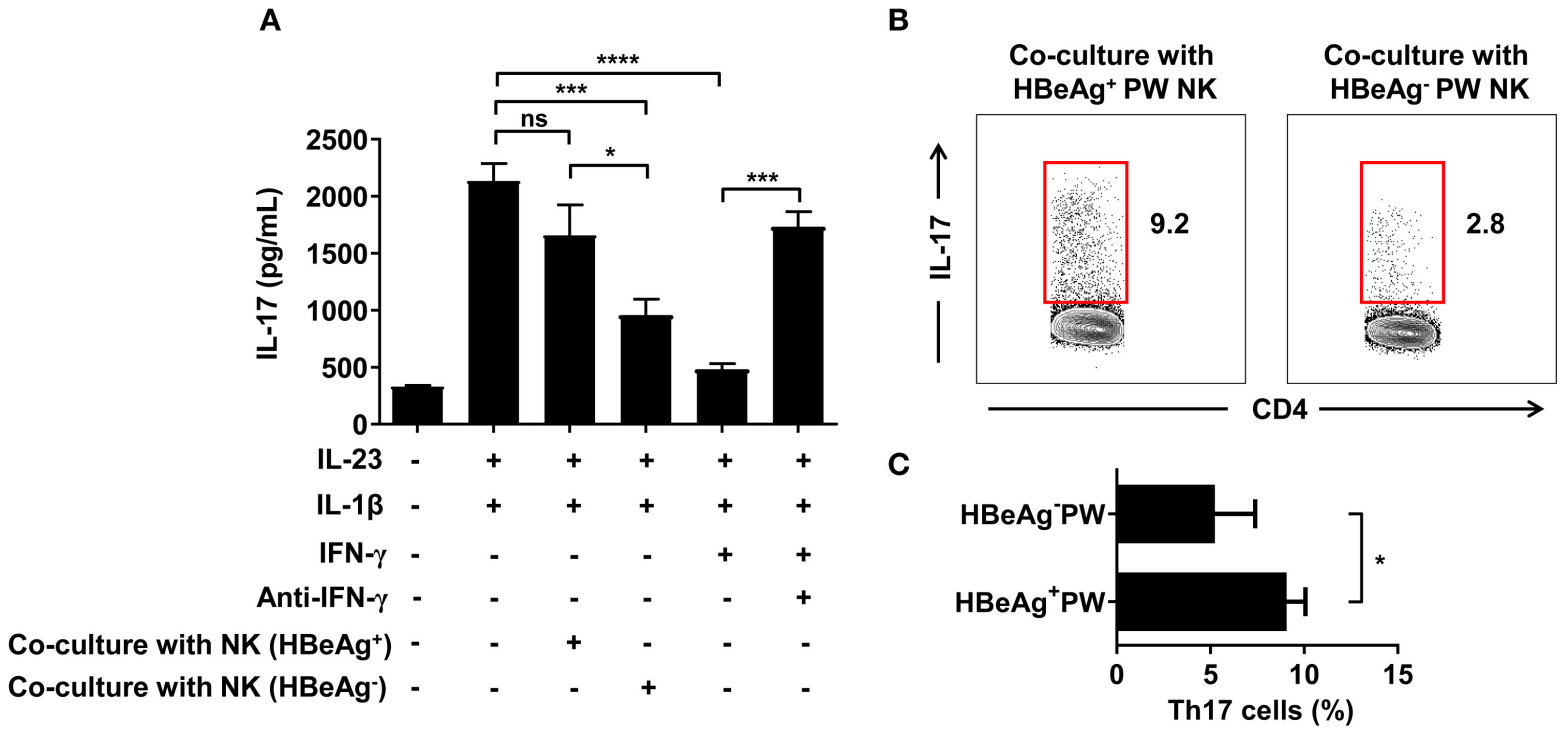
NK cells derived from HBeAg^+^ pregnant women with hepatitis B failed to inhibit Th17 cell polarization. **(A)** NK cells from HBeAg^-^ or HBeAg^+^ pregnant women were co-cultured with CD4^+^ T cells. IFN-γ or IFN-γ neutralizing antibodies were added to the IFN-γ blocking experiment, and cytokines (IL-23 and IL-1β) promoting Th17 polarization were added in the IFN-γ blocking experiment. IL-17 was detected in the culture supernatant under different co-culture conditions. The first column was the negative control; the second column was with the addition of IL-1β and IL-23; the third column was with the addition of IL-1β, IL-23, and NK cells from HBeAg^+^ pregnant women; the fourth column was with the addition of IL-1β, IL-23, and NK cells from HBeAg^-^ pregnant women; the fifth column was with the addition of IL-1β, IL-23, and IFN-γ; and the sixth column was with the addition of IL-1β, IL-23, IFN-γ, and IFN-γ neutralizing antibody. **(B)** Representative dot plots represent the proportion of Th17 cells in the co-culture conditions of NK cells and CD4^+^ T cells from HBeAg^-^ or HBeAg^+^ pregnant women. **(C)** Pooled data showing the proportion of Th17 cells in the co-culture conditions. Paired two-tailed Student’s *t*-tests were used. *P<0.05; ***P<0.001; ****P<0.0001; n.s., not significant.

To verify whether NK cells could inhibit Th17 cell polarization by IFN-γ, we conducted antibody blocking assay. In the antibody blocking experiment, we used magnetic beads to separate CD4^+^ T cells from different pregnant women and cultured Th17 cells by polarization *in vitro*. After adding IL-1β and IL-23 followed by IFN-γ and IFN-γ neutralizing antibodies to the culture system, we detected IL-17 levels using ELISA. The results showed that the level of IL-17 in the culture supernatant of the IFN-γ group was significantly lower than that of the control group, confirming that IFN-γ could inhibit the polarization of Th17 cells. The level of IL-17 in the supernatant of the IFN-γ neutralizing antibody group was significantly higher than that of the IFN-γ group, indicating that the polarization of Th17 cells was restored after IFN-γ was neutralized by the antibody, confirming that the inhibition of Th17 cell polarization was the effect of IFN-γ treatment. When we co-cultured NK cells from HBeAg^+^ and HBeAg^-^ pregnant women with CD4^+^ T cells and measured the IL-17 levels in culture supernatant by ELISA, we found that the IL-17 level in the culture supernatant of the HBeAg^-^ pregnant women was significantly lower than that of the control group and HBeAg^+^ pregnant women. These results confirmed that only the NK cells derived from HBeAg^-^ pregnant women significantly inhibited Th0 cell polarization to Th17 cells, while NK cells derived from HBeAg^+^ pregnant women did not inhibit Th17 cell polarization (**Figure 7A**). When we detected the proportion of Th17 cells in the co-cultured conditions by flow cytometry, we found that the proportion of Th17 cells from the HBeAg^-^ pregnant women was significantly lower than that from the HBeAg^+^ pregnant women (**Figures 7B, C**). Taken together, these findings indicate that NK cells derived from HBeAg^+^ pregnant women have a significantly lower inhibitory effect on Th17 cell polarization.

## 4 Discussion

HBV reactivation may occur in pregnant women during the inactive phase of HBV infection, leading to deterioration of liver function and endangering the health of both the mother and the fetus (Borgia et al., 2012). This process is likely caused by abnormal regulation of the immune system. On the one hand, the immune system needs to control the excessive replication of HBV and exert immune defense functions during pregnancy (Meier et al., 2021); on the other hand, as the embryo acts as a semi-allogeneic antigen, the maternal immune system needs to maintain local immune tolerance to sustain immune homeostasis (Alippe et al., 2025). NK cells also play a crucial role in decidual formation and uterine vascular remodeling, maintaining important immune tolerance functions in normal pregnancy (Jabrane-Ferrat and Siewiera, 2014). During viral infections in pregnancy, they secrete IFN-γ to inhibit viral replication or participate in antiviral immunity by directly killing virus-infected hepatocytes (Tang et al., 2024). Therefore, NK cells play a significant role in the occurrence, development, and outcome of CHB during pregnancy.

In addition to classical cytotoxic functions, NK cells also exert immunomodulatory effects through cytokine secretion. Previous studies have shown that compared with those of healthy individuals, the levels of IFN-γ and TNF-α secreted by all NK cells, CD56^bright^ NK subsets, and CD56^dim^ NK subsets of CHB patients are significantly decreased (Oliviero et al., 2009; Lunemann et al., 2014). Similarly, we found that the IFN-γ levels secreted by all NK cells, CD56^bright^ NK subsets, and CD56^dim^ NK subsets in HBeAg^+^ pregnant women were lower than those in HBeAg^-^ pregnant women and healthy pregnant women, consistent with the plasma IFN-γ levels. These findings indicate that the exacerbation of hepatitis in HBeAg^+^ pregnant women is not only accompanied by changes in NK cell subsets but also by weakened IFN-γ secretion, which might affect the functions of other immune cells. Studies have shown that the number of Th17 cells in the blood of HBV-infected patients is significantly higher than that in healthy controls, and IL-17 exacerbates liver injury in hepatitis patients, correlating with liver fibrosis (Piaserico et al., 2019; Li et al., 2021). IL-17 can mobilize neutrophils to induce pro-inflammatory cytokine production or induce fibroblasts to express intercellular adhesion molecule-1 (ICAM-1), promote T cell proliferation, and exacerbate inflammatory responses (Torchinsky and Blander, 2010). We found that HBeAg^+^ pregnant women had more Th17 cells and higher plasma IL-17 levels than HBeAg^-^ pregnant women. These phenomena suggest that the exacerbation of hepatitis B during pregnancy may be associated with increased numbers of Th17 cells and excessive IL-17 secretion participating in inflammatory responses.

CD4^+^ T cells are activated and then proliferate and differentiate into different T cell subsets after receiving dual-signal stimulation from antigen-presenting cells. Cytokines such as IL-6, TGF-β, and IL-23 can induce Th0 cells to differentiate into Th17 cells. To date, multiple cytokines have been found to promote or inhibit Th17 cell differentiation, among which IFN-γ can inhibit Th17 cell generation by interfering with TGF-β receptors (Takimoto et al., 2010). Whether IFN-γ from NK cells affects the polarization of Th17 cells during the exacerbation of hepatitis B in pregnancy remains unclear. In this study, adding IFN-γ or IFN-γ neutralizing antibodies to Th17 cell polarization culture systems showed that IFN-γ inhibited Th17 cell polarization. Co-culturing CD4^+^ T cells with NK cells from different pregnant women revealed that only NK cells from HBeAg^-^ pregnant women effectively inhibited Th0 cell polarization into Th17 cells, while NK cells from HBeAg^+^ pregnant women failed to inhibit Th17 cell polarization. These results confirm that insufficient IFN-γ production by NK cells cannot effectively suppress Th17 cell polarization in HBeAg^+^ pregnant women. In addition to IFN-γ from NK cells, other cytokines in HBeAg^+^ pregnant women may influence Th17 cell polarization. Studies have shown that plasma IL-24 levels are elevated in patients with CHB and HBV-associated hepatocellular carcinoma, and *in vitro* experiments confirm that IL-24 reduces Th17 cell proportion and IL-17 expression in liver-infiltrating lymphocytes and inhibits Th17 cell polarization through the NF-κB pathway (Zhang et al., 2022b). A study of patients with fungal infection found that dectin-1 induces TGF-β expression by regulating IFN-β, thereby enhancing non-pathogenic Th17 cell polarization (Gringhuis et al., 2022). Understanding the relationship between Th17 cells and various conditions is important, and understanding the interplay of NK cells and Th17 cells in the context of pregnancy in women with hepatitis B is essential.

There are several limitations to this study that should be acknowledged. First, we conducted our study at a single center and examined a relatively small number of patients, which might have affected the reliability of the results. Second, we could not fully explore the mechanism by which IFN-γ derived from NK cells affects Th17 cell polarization, and it should therefore be further explored in further research. Third, due to the lack of access to liver specimens from pregnant women with hepatitis B, it remains unclear whether such a mechanism occurs locally in the liver. Fourth, we studied a limited number of factors that impact Th17 polarization in pregnant women with hepatitis B, and other factors that may also impact Th17 polarization should be explored.

In conclusion, our work found that the exacerbation of hepatitis B during pregnancy mainly occurs in HBeAg^+^ pregnant women, potentially due to an increased number of CD56^dim^ NK cells, which is associated with a high expression of activating receptors NKG2D and CD226 and a reduced expression of inhibitory receptors NKG2A and CD158b, enhancing cytotoxicity. Moreover, a decreased number of CD56^bright^ NK cells and a reduction in IFN-γ production from total NK cells lead to a failure in effectively inhibiting Th17 cell polarization. This failure increases the IL-17 levels, thereby exacerbating hepatic inflammation. Collectively, these findings advance our understanding of the pathogenesis and mechanisms of exacerbation of hepatitis B during pregnancy. Using these findings to intervene in NK cell functions through appropriate NK-cell-related targeted therapy may alleviate or prevent the exacerbation of hepatitis B during pregnancy.

## Data Availability

The original contributions presented in the study are included in the article/**Supplementary Material**. Further inquiries can be directed to the corresponding author.

## References

[B1] Alippe Y. Hatterschide J. Coyne C. B. Diamond M. S. (2025). Innate immune responses to pathogens at the maternal-fetal interface. Nat. Rev. Immunol. doi: 10.1038/s41577-025-01191-0, PMID: 40533582

[B2] Borgia G. Carleo M. A. Gaeta G. B. Gentile I. (2012). Hepatitis B in pregnancy. World J. Gastroenterol. 18, 4677–4683. doi: 10.3748/wjg.v18.i34.4677, PMID: 23002336 PMC3442205

[B3] Cooper M. A. Fehniger T. A. Turner S. C. Chen K. S. Ghaheri B. A. Ghayur T.. (2001). Human natural killer cells: a unique innate immunoregulatory role for the CD56(bright) subset. Blood 97, 3146–3151. doi: 10.1182/blood.v97.10.3146, PMID: 11342442

[B4] Easterbrook P. J. Luhmann N. Bajis S. Min M. S. Newman M. Lesi O.. (2024). WHO 2024 hepatitis B guidelines: an opportunity to transform care. Lancet Gastroenterol. Hepatol. 9, 493–495. doi: 10.1016/S2468-1253(24)00089-X, PMID: 38614110

[B5] Fu B. Li X. Sun R. Tong X. Ling B. Tian Z.. (2013). Natural killer cells promote immune tolerance by regulating inflammatory TH17 cells at the human maternal-fetal interface. Proc. Natl. Acad. Sci. U.S.A. 110, E231–E240. doi: 10.1073/pnas.1206322110, PMID: 23271808 PMC3549088

[B6] Glassner A. Eisenhardt M. Kramer B. Korner C. Coenen M. Sauerbruch T.. (2012). NK cells from HCV-infected patients effectively induce apoptosis of activated primary human hepatic stellate cells in a TRAIL-, FasL- and NKG2D-dependent manner. Lab. Invest. 92, 967–977. doi: 10.1038/labinvest.2012.54, PMID: 22449797

[B7] Gringhuis S. I. Kaptein T. M. Remmerswaal E. B. M. Drewniak A. Wevers B. A. Theelen B.. (2022). Fungal sensing by dectin-1 directs the non-pathogenic polarization of T(H)17 cells through balanced type I IFN responses in human DCs. Nat. Immunol. 23 (12):1735-1748. doi: 10.1038/s41590-022-01348-2, PMID: 36456734 PMC9747615

[B8] Hanna J. Goldman-Wohl D. Hamani Y. Avraham I. Greenfield C. Natanson-Yaron S.. (2006). Decidual NK cells regulate key developmental processes at the human fetal-maternal interface. Nat. Med. 12, 1065–1074. doi: 10.1038/nm1452, PMID: 16892062

[B9] Jabrane-Ferrat N. Siewiera J. (2014). The up side of decidual natural killer cells: new developments in immunology of pregnancy. Immunology 141, 490–497. doi: 10.1111/imm.12218, PMID: 24256296 PMC3956423

[B10] Joshi S. S. Coffin C. S. (2020). Hepatitis B and pregnancy: virologic and immunologic characteristics. Hepatol. Commun. 4, 157–171. doi: 10.1002/hep4.1460, PMID: 32025602 PMC6996345

[B11] Lentz L. S. Stutz A. J. Meyer N. Schubert K. Karkossa I. von Bergen M.. (2022). Human chorionic gonadotropin promotes murine Treg cells and restricts pregnancy-harmful proinflammatory Th17 responses. Front. Immunol. 13. doi: 10.3389/fimmu.2022.989247, PMID: 36203576 PMC9531259

[B12] Li N. Yamamoto G. Fuji H. Kisseleva T. (2021). Interleukin-17 in liver disease pathogenesis. Semin. Liver Dis. 41, 507–515. doi: 10.1055/s-0041-1730926, PMID: 34130335

[B13] Lunemann S. Malone D. F. Hengst J. Port K. Grabowski J. Deterding K.. (2014). Compromised function of natural killer cells in acute and chronic viral hepatitis. J. Infect. Dis. 209, 1362–1373. doi: 10.1093/infdis/jit561, PMID: 24154737

[B14] Meier M. A. Calabrese D. Suslov A. Terracciano L. M. Heim M. H. Wieland S. (2021). Ubiquitous expression of HBsAg from integrated HBV DNA in patients with low viral load. J. Hepatol. 75, 840–847. doi: 10.1016/j.jhep.2021.04.051, PMID: 34004216

[B15] Michel T. Poli A. Cuapio A. Briquemont B. Iserentant G. Ollert M.. (2016). Human CD56bright NK cells: an update. J. Immunol. 196, 2923–2931. doi: 10.4049/jimmunol.1502570, PMID: 26994304

[B16] Moretta L. Bottino C. Pende D. Mingari M. C. Biassoni R. Moretta A. (2002). Human natural killer cells: their origin, receptors and function. Eur. J. Immunol. 32, 1205–1211. doi: 10.1002/1521-4141(200205)32:5<1205::Aid-immu1205>3.0.Co;2-y 11981807

[B17] Oliviero B. Varchetta S. Paudice E. Michelone G. Zaramella M. Mavilio D.. (2009). Natural killer cell functional dichotomy in chronic hepatitis B and chronic hepatitis C virus infections. Gastroenterology 137, 1151–1160. doi: 10.1053/j.gastro.2009.05.047, PMID: 19470388

[B18] Pawlowska M. (2024). Hepatitis B virus infections in pregnant women and children in the era of HBV elimination. Clin. Exp. Hepatol. 10, 227–231. doi: 10.5114/ceh.2024.145364, PMID: 40290526 PMC12022621

[B19] Piaserico S. Messina F. Russo F. P. (2019). Managing psoriasis in patients with HBV or HCV infection: practical considerations. Am. J. Clin. Dermatol. 20, 829–845. doi: 10.1007/s40257-019-00457-3, PMID: 31222626

[B20] Plug A. Barenbrug L. Moerings B. G. J. de Jong E. M. G. van der Molen R. G. (2025). Understanding the role of immune-mediated inflammatory disease related cytokines interleukin 17 and 23 in pregnancy: A systematic review. J. Transl. Autoimmun. 10, 100279. doi: 10.1016/j.jtauto.2025.100279, PMID: 40035074 PMC11874717

[B21] Takimoto T. Wakabayashi Y. Sekiya T. Inoue N. Morita R. Ichiyama K.. (2010). Smad2 and Smad3 are redundantly essential for the TGF-beta-mediated regulation of regulatory T plasticity and Th1 development. J. Immunol. 185, 842–855. doi: 10.4049/jimmunol.0904100, PMID: 20548029

[B22] Tang Q. Wang C. Li H. Chen Z. Liu X. Xue Y.. (2024). PgRNA closely correlates to cytokine profile in HBeAg-positive pregnant women undergoing prophylactic antiviral intervention. Front. Immunol. 15. doi: 10.3389/fimmu.2024.1511855, PMID: 39720709 PMC11668345

[B23] Tao Y. Li Y. H. Piao H. L. Zhou W. J. Zhang D. Fu Q.. (2015). CD56(bright)CD25+ NK cells are preferentially recruited to the maternal/fetal interface in early human pregnancy. Cell Mol. Immunol. 12, 77–86. doi: 10.1038/cmi.2014.26, PMID: 24793405 PMC4654367

[B24] Torchinsky M. B. Blander J. M. (2010). T helper 17 cells: discovery, function, and physiological trigger. Cell Mol. Life Sci. 67, 1407–1421. doi: 10.1007/s00018-009-0248-3, PMID: 20054607 PMC11115816

[B25] Tran T. T. (2016). Hepatitis B in pregnancy. Clin. Infect. Dis. 62 Suppl 4, S314–S317. doi: 10.1093/cid/ciw092, PMID: 27190321 PMC4889900

[B26] Xie H. Zeng J. Yan X. Shen N. Zheng X. Luo H. (2022). Clinical significance and properties of IFN-γ+IL-17+ Th17 cells in liver injury associated with chronic hepatitis B virus infection. Digestion 103, 438–450. doi: 10.1159/000526924, PMID: 36265446

[B27] Zhang H. Yan X. Yang C. Zhan Q. Fu Y. Luo H.. (2020). Intrahepatic T helper 17 cells recruited by hepatitis B virus X antigen-activated hepatic stellate cells exacerbate the progression of chronic hepatitis B virus infection. J. Viral Hepat. 27, 1138–1149. doi: 10.1111/jvh.13352, PMID: 32559002

[B28] Zhang L. Jiang T. Yang Y. Deng W. Lu H. Wang S.. (2022a). Postpartum hepatitis and host immunity in pregnant women with chronic HBV infection. Front. Immunol. 13. doi: 10.3389/fimmu.2022.1112234, PMID: 36685527 PMC9846060

[B29] Zhang M. Zhao H. Gao H. (2022b). Interleukin-24 limits tumor-infiltrating T helper 17 cell response in patients with hepatitis B virus-related hepatocellular carcinoma. Viral Immunol. 35, 212–222. doi: 10.1089/vim.2021.0174, PMID: 35099297

