# Supplementary material for: Alterations in peripheral blood NK cell subsets and function in patients with HBeAg-positive chronic hepatitis B during pregnancy
Source: Front Cell Infect Microbiol. 2025 Oct 6;15:1657367. doi: 10.3389/fcimb.2025.1657367 (PMC12535973; doi:10.3389/fcimb.2025.1657367)
Supplement: Supplementary file 1 [file Table1.docx]

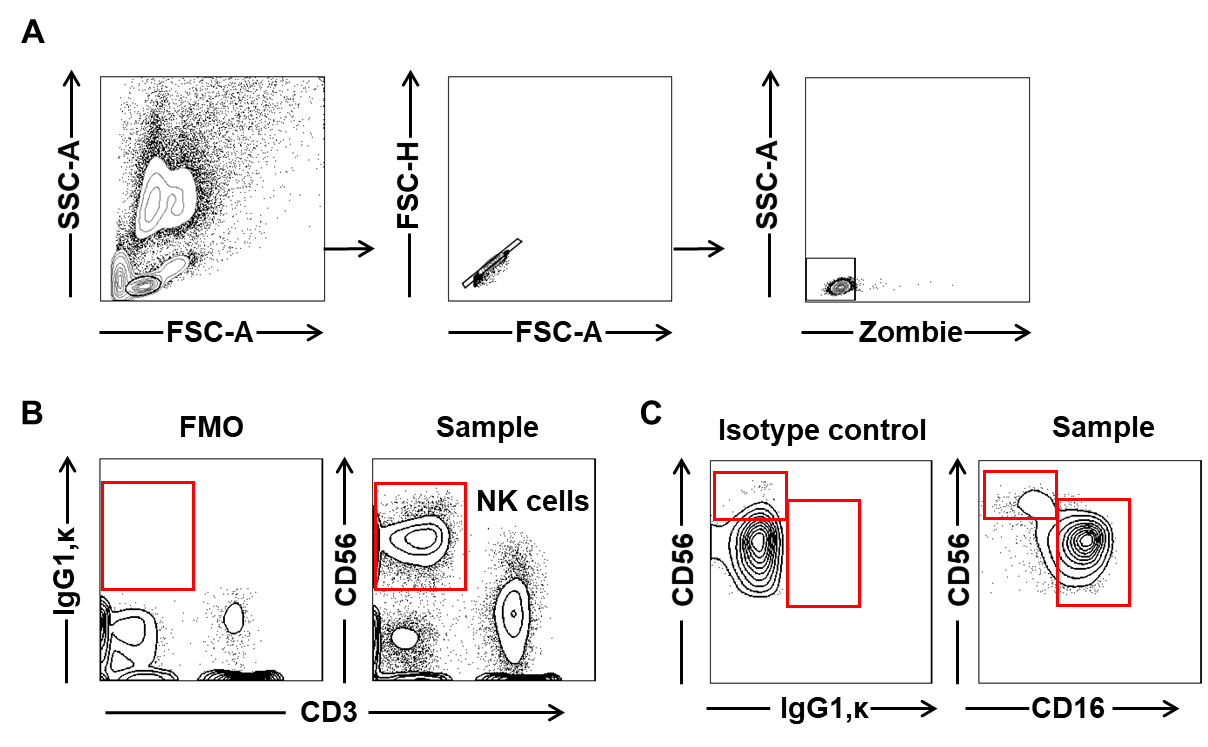


**Supplementary Figure 1. The gating strategy for detecting NK cells and their subsets in peripheral blood by flow cytometry.** Total lymphocytes were gated by FSC-A and SSC-A, followed by gating of single cells using FSC-A and FSC-H, and dead cells were excluded by Zombie staining (Supplementary Figure 1A). Total NK cells were gated by FMO control (Supplementary Figure 1B). Within the NK cell gate, CD56^bright^CD16^-^ NK cells and CD56^dim^CD16^+^ NK cells were gated by isotype control (Supplementary Figure 1C).


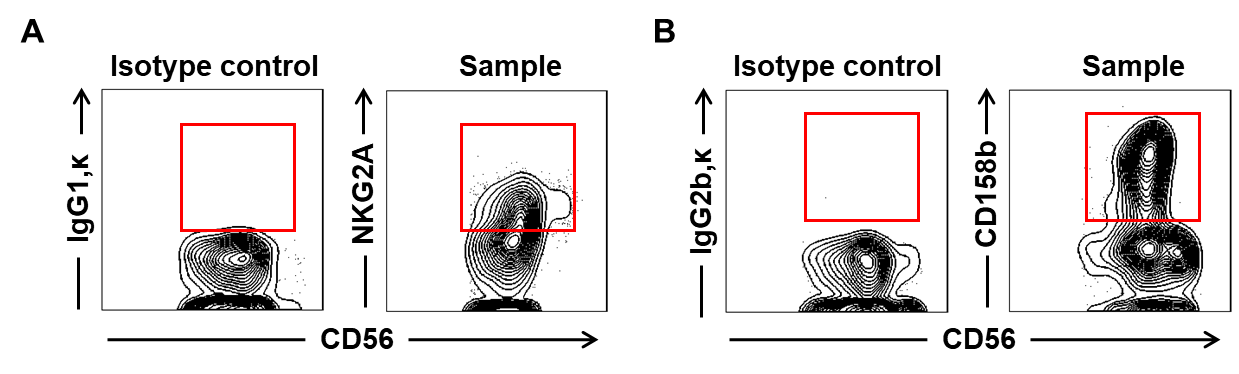


**Supplementary Figure 2. The gating strategy for isotype controls and experimental groups of NKG2A and CD158b on the surface of NK cells.**


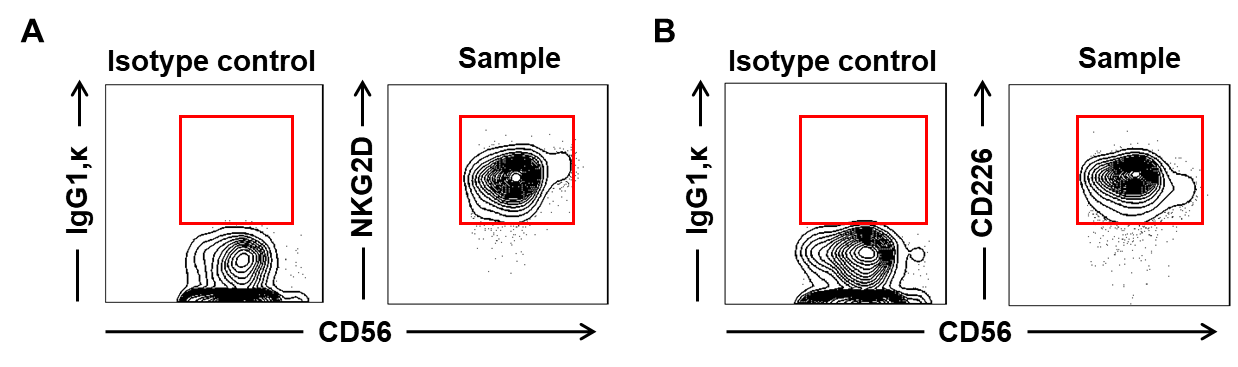


**Supplementary Figure 3. The gating strategy for isotype controls and experimental groups of NKG2D and CD226 on the surface of NK cells.**

**
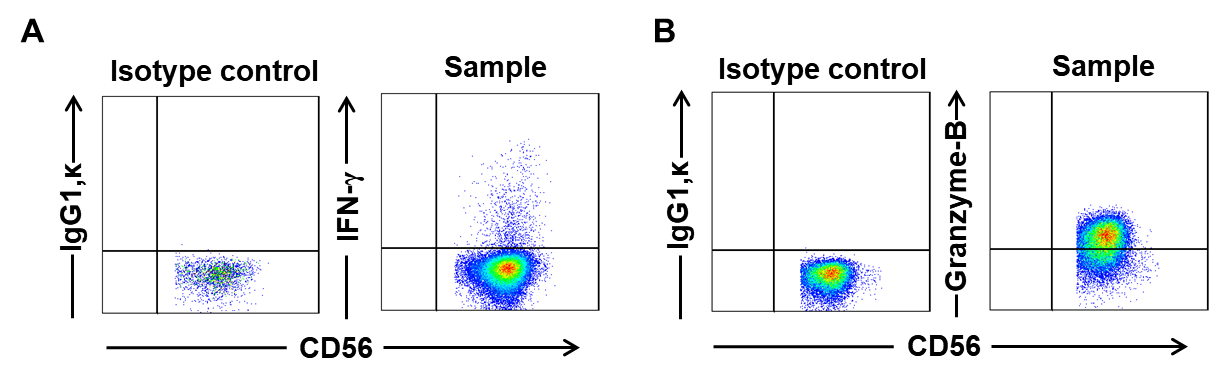
**

**Supplementary Figure 4. The gating strategy for isotype controls and experimental groups of IFN-γ within NK cells.**

**
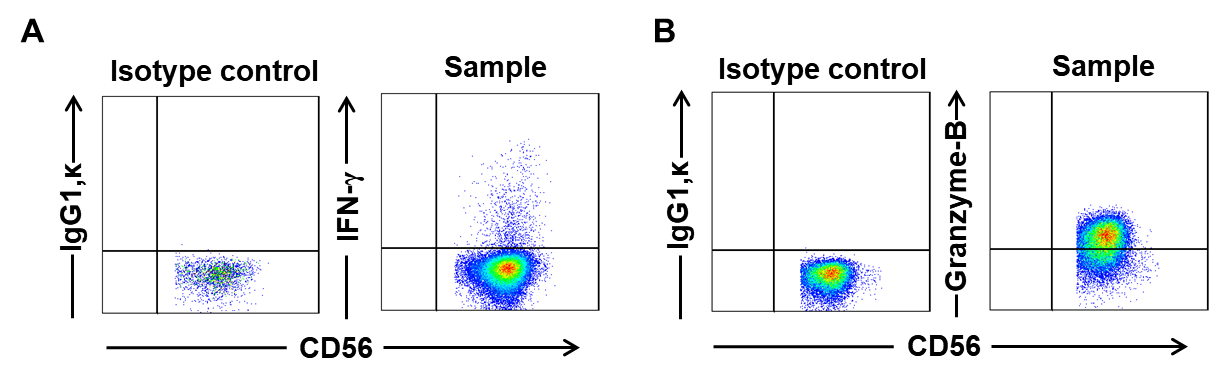
**

**Supplementary Figure 5. The gating strategy for isotype controls and experimental groups of granzyme-B within NK cells.**
